# Oral Vaccination with Attenuated *Salmonella typhimurium*-Delivered *Ts*Pmy DNA Vaccine Elicits Protective Immunity against *Trichinella spiralis* in BALB/c Mice

**DOI:** 10.1371/journal.pntd.0004952

**Published:** 2016-09-02

**Authors:** Lei Wang, Xiaohuan Wang, Kuo Bi, Ximeng Sun, Jing Yang, Yuan Gu, Jingjing Huang, Bin Zhan, Xinping Zhu

**Affiliations:** 1 Department of Medical Microbiology and Parasitology, School of Basic Medical Sciences, Capital Medical University, Beijing, PR China; 2 Beijing Tropical Medicine Research Institute, Beijing Friendship Hospital, Capital Medical University, Beijing, PR China; 3 Department of Pediatrics, National School of Tropical Medicine, Baylor College of Medicine, Houston, Texas, United States of America; McGill University, CANADA

## Abstract

**Background:**

Our previous studies showed that *Trichinella spiralis* paramyosin (*Ts*Pmy) is an immunomodulatory protein that inhibits complement C1q and C8/C9 to evade host complement attack. Vaccination with recombinant *Ts*Pmy protein induced protective immunity against *T*. *spiralis* larval challenge. Due to the difficulty in producing *Ts*Pmy as a soluble recombinant protein, we prepared a DNA vaccine as an alternative approach in order to elicit a robust immunity against *Trichinella* infection.

**Methods and Findings:**

The full-length *Ts*Pmy coding DNA was cloned into the eukaryotic expression plasmid pVAX1, and the recombinant pVAX1/*Ts*Pmy was transformed into attenuated *Salmonella typhimurium* strain SL7207. Oral vaccination of mice with this attenuated *Salmonella*-delivered *Ts*Pmy DNA vaccine elicited a significant mucosal sIgA response in the intestine and a systemic IgG antibody response with IgG2a as the predominant subclass. Cytokine analysis also showed a significant increase in the Th1 (IFN-γ, IL-2) and Th2 (IL-4, 5, 6, 10) responses in lymphocytes from the spleen and MLNs of immunized mice upon stimulation with *Ts*Pmy protein. The expression of the homing receptors CCR9/CCR10 on antibody secreting B cells may be related to the translocation of IgA-secreted B cells to local intestinal mucosa. The mice immunized with *Salmonella*-delivered *Ts*Pmy DNA vaccine produced a significant 44.8% reduction in adult worm and a 46.6% reduction in muscle larvae after challenge with *T*. *spiralis* larvae.

**Conclusion:**

Our results demonstrated that oral vaccination with *Ts*Pmy DNA delivered by live attenuated *S*. *typhimurium* elicited a significant local IgA response and a mixed Th1/Th2 immune response that elicited a significant protection against *T*. *spiralis* infection in mice.

## Author Summary

Trichinellosis is one of the most important food-borne parasitic zoonoses, and a serious public health issue worldwide. Developing a vaccine is an alternative approach to control the disease. *Ts*Pmy is a paramyosin expressed by *Trichinella spiralis* to bind and neutralize human complement and a vaccine antigen. We made a DNA vaccine of *Ts*Pmy orally delivered by attenuated *Salmonella typhimurium* that elicited a robust Th1/Th2 and mucosa IgA responses, and protected mice against *T*. *spiralis* infection with significant worm reduction against larval challenge. The attenuated *Salmonella*-delivered *Ts*Pmy DNA vaccine provides a feasible and promising approach for controlling trichinellosis in human and domestic animals.

## Introduction

Trichinellosis, a serious food-borne parasitic zoonosis and an important public health problem worldwide, is mainly caused by infection with the tissue-dwelling nematode *Trichinella spiralis* [1, 2]. People develop this infection through ingestion of raw or undercooked meat contaminated with encapsulated parasite larva. Domestic pork has been the major source of this infection in China and other countries. Due to the increased consumption of pork and other meat, trichinellosis is an emerging or re-emerging disease in many countries [2]. In China, 17 outbreaks of human trichinellosis were reported, with 828 cases and 11 deaths in eight provinces between 2000 and 2003 [3]. The development of vaccine(s) has become an urgent need for controlling trichinellosis in human and domestic animals.

*T*. *spiralis* is an intestinal parasite whereby the adult worm dwells in the small intestine with the head embedding into the mucosa and the epithelial layer. Female worms produce newborn larvae that penetrate into the intestinal wall and migrate through the blood circulation to the muscle tissue where they form cysts. Obviously, the intestinal mucosa becomes the site for parasite host interaction and the first barrier for protecting the host against *Trichinella* infection [4]. Therefore, the local mucosal immune response is crucial for establishing protective immunity against intestinal parasite such as *T*. *spiralis*.

Other studies have shown that attenuated *Salmonella typhimurium* is an effective oral delivery vector for heterologous antigens to induce long-lasting mucosal and systemic immune responses against infections with intestinal pathogens, providing an efficient design for novel vaccination strategies [5, 6]. This novel delivery system has proven successful in inducing protective immunity against many infections such as *Toxoplasma gondii*[7], *Giardia lamblia*[8] and *Trypanosoma cruzi*[9]. In our previous study, attenuated *Salmonella typhimurium* was used to orally deliver a DNA vaccine of *Ts*87, an excretory/secretory antigen from *T*. *spiralis*, which has shown significant protection against *T*. *spiralis* larval challenge in a mouse model [10]. Additional evidence has shown that attenuated bacteria could effectively induce a mucosal immune response and enhance antibody secreting cells (ASCs) homing to the epithelium of the intestine. The secretory IgA (sIgA) in the mucosal immune response plays important roles in killing or expelling intestinal pathogens [11, 12]. In this study, we developed a new DNA vaccine targeting *Ts*Pmy, the paramyosin protein of *T*. *spiralis* that induced protective immunity when recombinant protein was used [13], that was delivered by attenuated *S*. *typhimurium*. Mice orally vaccinated with *Salmonella*-delivered *Ts*Pmy DNA vaccine elicited robust mucosal and systemic immune responses that induced a significantly protective immunity against *T*. *spiralis* larval challenge.

## Materials and Methods

### Ethics statement

Female BALB/c 6–8 weeks old mice were provided by Laboratory Animal Services Centre of Capital Medical University. Mice were raised under specific pathogen-free conditions with suitable temperature and humidity. All experimental procedures were reviewed and approved by the Capital Medical University Animal Care and Use Committee (approval number: 2012-X-108) and complied with the NIH Guide for the Care and Use of Laboratory Animals.

### Bacteria

The attenuated *S*. *typhimurium* SL7207 strain that could deliver heterologous antigens with the virulent gene aroA knockout and was not pathogenic to mice via oral administration was kindly provided by Prof. J.S. He of Beijing Jiaotong University.

### Plasmid construction and transformation into *S*. *typhimurium* SL7207

The full-length DNA encoding for *Ts*Pmy (accession number: EF429310) was amplified from *T*. *spiralis* total cDNA using the following primers: forward, 5’-CGGGATCCATGTCTCTGTATCG CAGTCCCAGT-3’ and reverse 5’-CGGAATTCATATTCATGTCCTTCT TCCATCAC-3’. The amplified DNA fragment was cloned into the eukaryotic expression vector pVAX1 (Invitrogen, USA) at the *BamHI* and *EcoRI* sites. The correct insert sequence and reading frame was confirmed by double-stranded DNA sequencing using the vector flanking primers T7 promoter and BGH reverse primer. The sequence-confirmed recombinant plasmid pVAX1-*Ts*Pmy and the empty plasmid pVAX1 were transformed into attenuated *S*. *typhimurium* strain SL7207 by electroporation, and the transformants were selected on LB agar plates containing 50μg/ml kanamycin and identified by PCR amplification with *Ts*Pmy specific primers and DNA sequencing.

### Parasites

The *T*. *spiralis* ISS 533 strain was maintained in female ICR mice. Each mouse was orally infected with 500 *T*. *spiralis* infective larvae. The adult worms were isolated from the intestines of infected mice at 5 days following larval challenge. The muscle larvae (ML) were recovered at 42 days post-infection from the muscle tissue of infected mice using a modified pepsin-hydrochloric acid digestion method [14].

### Preparation of recombinant *Ts*Pmy protein (r*Ts*Pmy)

A full-length cDNA encoding *Ts*Pmy was cloned into the expression vector pET-28a (+). The recombinant plasmid containing the *Ts*Pmy coding gene was transformed into *E*.*coli* BL21. The r*T*sPmy was expressed as insoluble inclusion body under induction of 1 mM IPTG and the urea-denatured r*Ts*Pmy was purified by Ni-affinity chromatography (Qiagen, USA) as previously described [15].

### *Ts*Pmy DNA immunization delivered by attenuated *S*. *typhimurium*

A total of 120 mice were randomly divided into three groups with 40 mice each. The first two groups were immunized orally with 1×10^8^ attenuated *S*. *typhimurium* SL7207 transformed with pVAX1-*Ts*Pmy (SL7207/pVAX1-*Ts*Pmy) or with empty pVAX1 (SL7207/pVAX1) in 100 μl of PBS. The third group of mice was given 100 μL of PBS only as control. All mice were given 100 μL of 10% NaHCO_3_ orally to neutralize gastric acids before oral inoculation with bacteria or PBS. All groups of mice were boosted twice with the same vaccine components at two weeks interval.

One week after each immunization, 5 mice from each group were sacrificed by CO_2_ inhalation. The serum, spleen, and mesenteric lymph nodes (MLNs) were collected to evaluate the levels of immune responses, and the intestines were collected with lavage fluid for measuring mucosal IgA. All mice were monitored by research personnel on a daily basis for general appearance, hunched posture, rough haircoat, labored breathing, lethargy, lameness, ataxia, diarrhea, abnormal vocalization and abnormal discharge from the eyes or nose. If any animal has bleeding diarrhea, labored breathing, severe leg injuries or become moribund it will be euthanized immediately by CO_2_ inhalation.

### Detection of *Ts*Pmy mRNA and recombinant *Ts*Pmy expression *in vivo*

One week after the first immunization, *Ts*Pmy mRNA was measured in the MLNs, spleen and liver tissues of immunized mice by reverse transcription polymerase chain reaction (RT-PCR) with the *Ts*Pmy-specific primers listed above. Total RNAs were isolated from the MLNs, spleen and liver tissues of immunized mice using TRIzol (Invitrogen, USA) according to the manufacturer’s instructions. First strand total cDNA was reversely transcribed from total RNAs using poly-T primer, and the *Tspmy* cDNA was amplified from the total cDNA using *Ts*Pmy specific primers. Mouse GAPDH cDNA was amplified from the same sample as a positive control. PCR products were detected by electrophoresis on 1% agarose gels.

To determine the expression of r*Ts*Pmy *in vivo*, MLNs of mice immunized with SL7207/pVAX1-*Ts*Pmy were fixed, frozen and cryosectioned. Tissue sections were washed three times with cold PBS and blocked with 5% normal goat serum (NGS, diluted in PBS, pH 7.6) at room temperature for 30 min. After incubation with anti-*Ts*Pmy monoclonal antibody 9G3[16] diluted 1:2000 in PBS plus 5% NGS at 4°Covernight, the tissue section was incubated with DyLight TM488-conjugated goat anti-mouse IgG at a 1:200 dilution. The MLN sections from mice receiving PBS only were incubated with the same antiserum as a negative control. The sections were examined and photographed using a fluorescence microscope (Leica, Germany).

### Detection of systemic and mucosal antibody response

Enzyme-linked immunosorbent assay (ELISA) was used to analyze the levels of antigen-specific IgG, IgG1 and IgG2a antibodies in the sera of the immunized mice as previously described [13]. The optical density (OD) at 450 nm was measured using an ELISA reader on sera diluted at 1:200.

To detect total or antigen-specific secretory IgA (sIgA) in intestinal washes, the intestinal lavage washes were prepared as described [17, 18]. Briefly, 10 cm of the small intestine beginning at the gastro-duodenal junction was cut for each sacrificed mouse, and the interior of the small intestine was flushed twice with 2 mL cold PBS. After centrifugation at 800×g for 10 min, the supernatants were harvested and stored at −80°C until use. Intestinal total sIgA was assessed using a sandwich-type ELISA by trapping intestinal mucosal IgA on a plate coated with purified rat anti-mouse IgA (BD Biosciences, USA), and the specific anti-*Ts*Pmy sIgA was measured by standard ELISA using r*Ts*Pmy coated plates as described[10].

### Cytokine testing

To measure the specific cellular immune responses in the lymphocytes isolated from the spleen and MLNs of immunized mice upon stimulation with r*Ts*Pmy, the production of IFN-γ, IL-2, IL-4, IL-5, IL-6 and IL-10 was detected using an ELISPOT assay according to the manufacturer’s instructions (BDTM ELISPOT, USA). Briefly, the mice were sacrificed one week after the third immunization, and the single lymphocyte suspensions were prepared by dissociating the spleen and MLNs through a mesh into the lymphocyte separation medium (Dakewe, China). The wells of plates were coated with the capture antibody (anti-mouse IFN-γ, IL-4, IL-6, and IL-10; BD Biosciences, USA) at 1: 200 dilutions in PBS and incubated overnight at 4°C. The plates were washed once with RPMI-1640 medium with 10% fetal bovine serum and blocked for 2 h at room temperature. A total of 1 × 10^6^ lymphocytes were added to each well in a total volume of 100 μL. The lymphocyte cells were stimulated with r*Ts*Pmy at a final concentration of 1 μg/mL at 37°C for 48 h in a 5% CO_2_ incubator. A total 100 μL of biotinylated detection antibody at 1: 200 in PBS containing 10% FBS was added into each well for 2 h. The wells were incubated with 100 μL of streptavidin-HRP for 1 h (BD Biosciences, USA) and developed with 100 μL of 3-amino-9-ethylcarbazole substrate solution for 30 s–5 min according to the manufacturer’s instruction. The spot-forming units (SFU) were counted automatically by a CTL ELISPOT reader and analyzed using ImmunoSpot image analyzer software v4.0.

### Detection of CCR9 and CCR10 on ASCs and expression of anti-*Ts*Pmy specific IgA in ASCs

To determine the expression of homing receptors (CCR9 and CCR10) on B cells induced by recombinant *Salmonella* inoculation, the mononuclear cells were isolated from spleens (SP), MLNs and intestinal *lamina propria* (LP) of mice two weeks after the third immunization as described [19–21]. ASCs were then enriched from these mononuclear cell suspensions using a magnetic bead B cell negative isolation kit (Invitrogen, USA). The enriched ASCs were blocked with rat anti-mouse CD16/CD32 mAb for 15 min (4°C) and incubated with anti-mouse CD19-FITC (BD Biosciences, San Diego, California), CD199(CCR9)-PE (BD Biosciences, USA), or CCR10-PerCP (R&D Systems, USA) mAbs or their isotype controls for surface marker staining for 30 min. After washing twice, cells were resuspended in 300 μL of 1% para-formaldehyde in PBS and analyzed by FACS (BD Biosciences, USA) to sort CCR9 and CCR10 expressed ASCs.

In order to determine if CCR9 expressing ASCs migrate toward chemokine CCL25, a total of 1×10^5^ ASCs were added to the upper chamber of a Transwell filter with 5 mm polycarbonate membrane (Costar Corning, USA) and allowed to migrate for 4 h at 37°C into the lower chamber containing RPMI-1640 supplemented with 25 ug/ml of CCL25 (Prospec, Israel). The cells that migrated into the lower chamber were counted and collected for detecting the expression of anti-*Ts*Pmy IgA using a modified ELISPOT assay. Briefly, wells coated with 1 μg/mL of r*Ts*Pmy were incubated with 1×10^5^ ASCs collected from the lower chamber. The cells expressing specific IgA against *Ts*Pmy was identified using biotin-conjugated anti-mouse IgA and Streptavidin-HRP.

### Challenge experiment

To evaluate the protective immunity, the left 20 mice of each group were each challenged with 500 *T*. *spiralis* muscle larvae two weeks after the third immunization. Adult worms were recovered from the intestines of 10 mice on day 5 post-infection, and the muscle larvae were recovered from muscle of ten mice 45 days after the challenge. The reduction evaluation in adult worm and muscle larvae was calculated based on the number of adult worms or muscle larvae collected from the group immunized with SL7207/pVAX1-*Ts*Pmy compared with those from the SL7207/pVAX1 control mice.

### Statistical analyses

Statistical analyses were performed with one-way ANOVA using SPSS version 17.0 software. All data were expressed as the mean ± standard deviation, with differences considered significant when *P* was less than 0.05.

## Results

### Transcription of *Ts*Pmy mRNA and recombinant *Ts*Pmy expression *in vivo*

**Fig 1:**
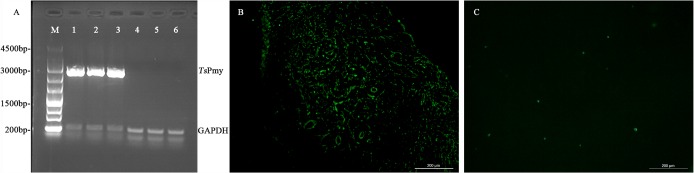
Expression of *Ts*Pmy in mice after the first immunization with SL7207/pVAX1-*Ts*Pmy. RT-PCR results (A) showed that the transcription of *Ts*Pmy mRNA was detected in the MLNs (lanes 1), spleen (lanes 2) and liver (lanes 3) of mice immunized with SL7207/pVAX1-*Ts*Pmy. No *Ts*Pmy mRNA was detected in the MLNs (lanes 4), spleen (lanes 5) and liver (lanes 6) of mice treated with SL7207/pVAX1 only. The expression of *Ts*Pmy protein was also detected in MLNs of mice immunized with SL7207/pVAX1-*Ts*Pmy using anti-*Ts*Pmy mAb 9G3 IFA (200×) (B). No fluorescence signal was detected in the sections of mice immunized with SL7207/pVAX1 only (200×) (C).

Total RNAs were isolated from the MLNs, liver and spleen tissues of mice one week after the first immunization for RT-PCR to determine the transcription of *Ts*Pmy in these tissues. The results showed that the *Ts*Pmy mRNA was transcribed in the tissues of mice immunized with SL7207/pVAX1-*Ts*Pmy but not in the mice received with SL7207/pVAX1 only (Fig 1A). Immunofluorescent staining with anti-*Ts*Pmy mAb 9G3 revealed that the *Ts*Pmy protein was expressed in MLNs of SL7207/pVAX1-*Ts*Pmy immunized mice (Fig 1B). No obvious fluorescence was observed in MLNs of mice treated with SL7207/pVAX1 (Fig 1C).

### Systemic and mucosal humoral immune responses induced by immunization with SL7207/pVAX1-*Ts*Pmy

**Fig 2:**
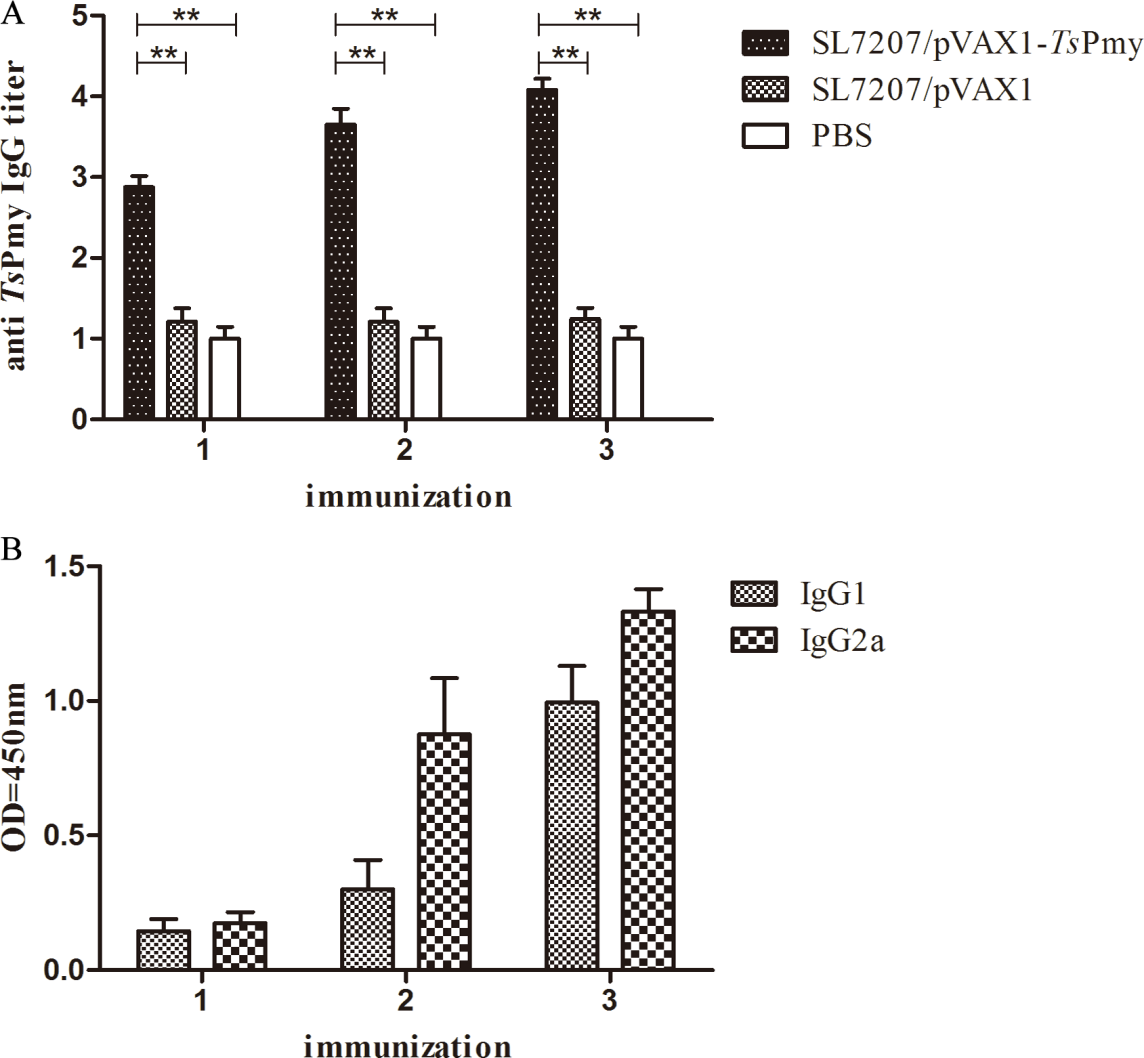
**Anti-*Ts*Pmy total IgG (A) and subclass IgG (B) responses in immunized mice was measured by ELISA.** Sera were collected one week after each immunization. The values shown for each group are the mean ± S.D. of antibody levels. The total IgG results represent log_10_ endpoint values for five individual mice, and the IgG1 and IgG2a levels are shown using OD value when the sera were diluted at 1:200. Double asterisks (**) indicate statistically significant differences between two groups (*p*< 0.01).

Serum samples of mice were collected one week after each immunization and the levels of specific anti-*Ts*Pmy IgG and its subclass (IgG1 and IgG2a) antibodies were measured by ELISA. A high titer of anti-*Ts*Pmy IgG was elicited following a boost with SL7207/pVAX1-*Ts*Pmy and reached their peak titer at one week after the third immunization. Nevertheless, none of the mice that received SL7207/pVAX1 or PBS orally showed detectable anti-*Ts*Pmy IgG responses (Fig 2A). The levels of anti-*Ts*Pmy IgG subclass IgG1 and IgG2a were also increased significantly in mice immunized with SL7207/pVAX1-*Ts*Pmy after the first boost and reached a peak after the second boost. The IgG2a level was significantly higher than IgG1 after the first boost, indicating that attenuated *Salmonella* delivered *Ts*Pmy DNA vaccine induced Th1/Th2-mixed type of immune response with Th1 being predominant (Fig 2B).

**Fig 3:**
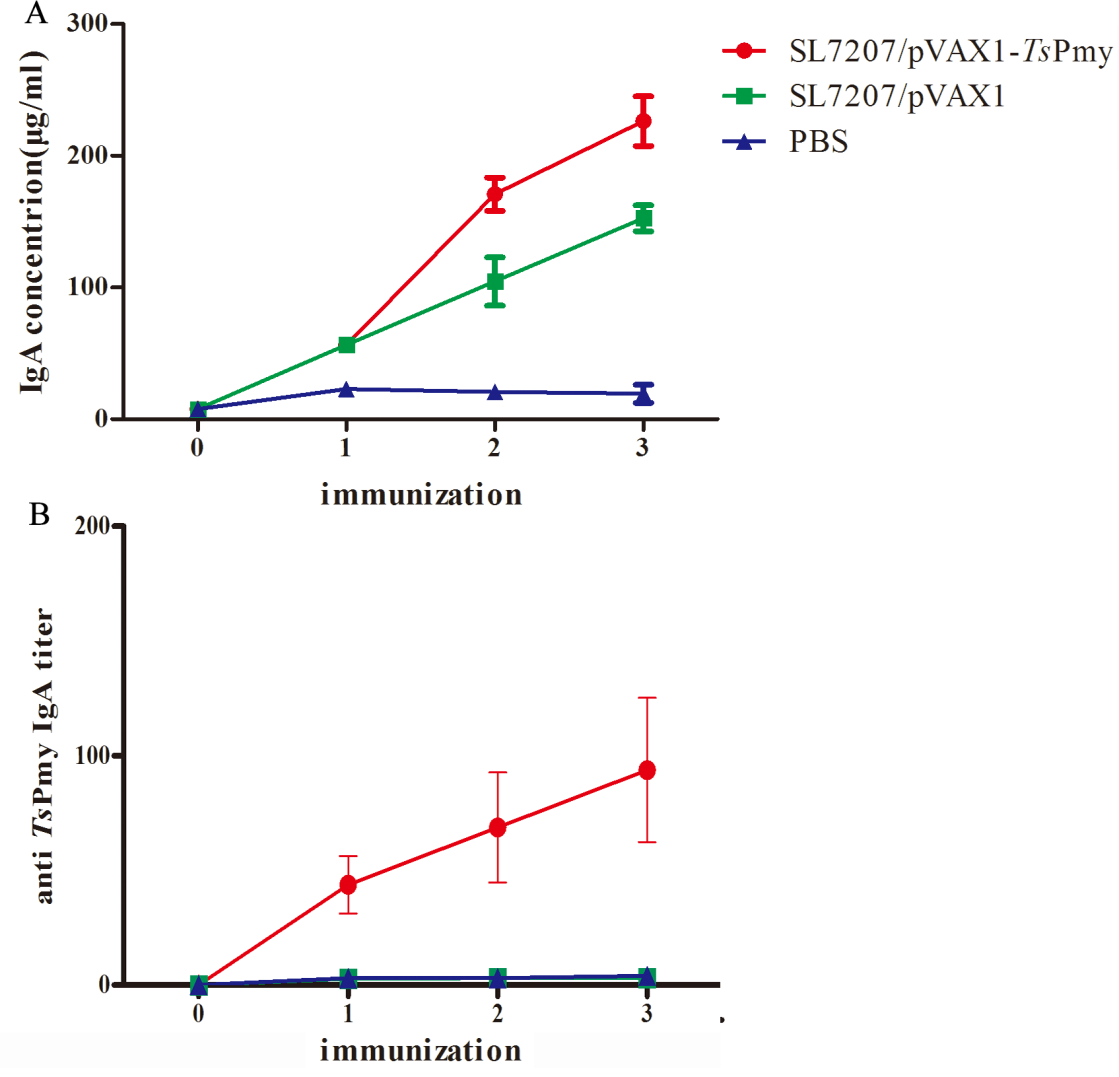
**The levels of total IgA (A) and anti-*Ts*Pmy-specific IgA (B) in intestinal washings of mice immunized with SL7207/pVAX1-*Ts*Pmy, vector control or PBS control.** The results are the mean ± S.D. for 5 mice per group.

To evaluate the intestinal mucosal sIgA response upon oral SL7207/pVAX1-*Ts*Pmy immunization, the total sIgA and anti-*Ts*Pmy specific sIgA were measured in intestinal mucosal washings by ELISA. Total sIgA level was significantly (*p*< 0.05) elevated in the intestinal mucosa of mice immunized with SL7207/pVAX1-*Ts*Pmy compared with those administered SL7207/pVAX1 or PBS (Fig 3A). The anti-*Ts*Pmy sIgA level was measured using a r*Ts*Pmy-coated plate. Anti-*Ts*Pmy specific sIgA was also significantly increased in the intestinal mucosa of mice immunized with SL7207/pVAX1-*Ts*Pmy compared with those treated with vector alone or PBS (Fig 3B).

### Cytokines profiles

To evaluate the cytokine profiles induced by SL7207/pVAX1-*Ts*Pmy immunization, 5 mice were sacrificed at 1 week after the third immunization. Spleen cells and MLN cells were collected and stimulated with 1 ug/ml of r*Ts*Pmy. Cytokines secreted by the lymphocytes including IFN-γ, IL-2, IL-4, IL-5, IL-6 and IL-10 were detected by ELISPOT assay.

**Fig 4:**
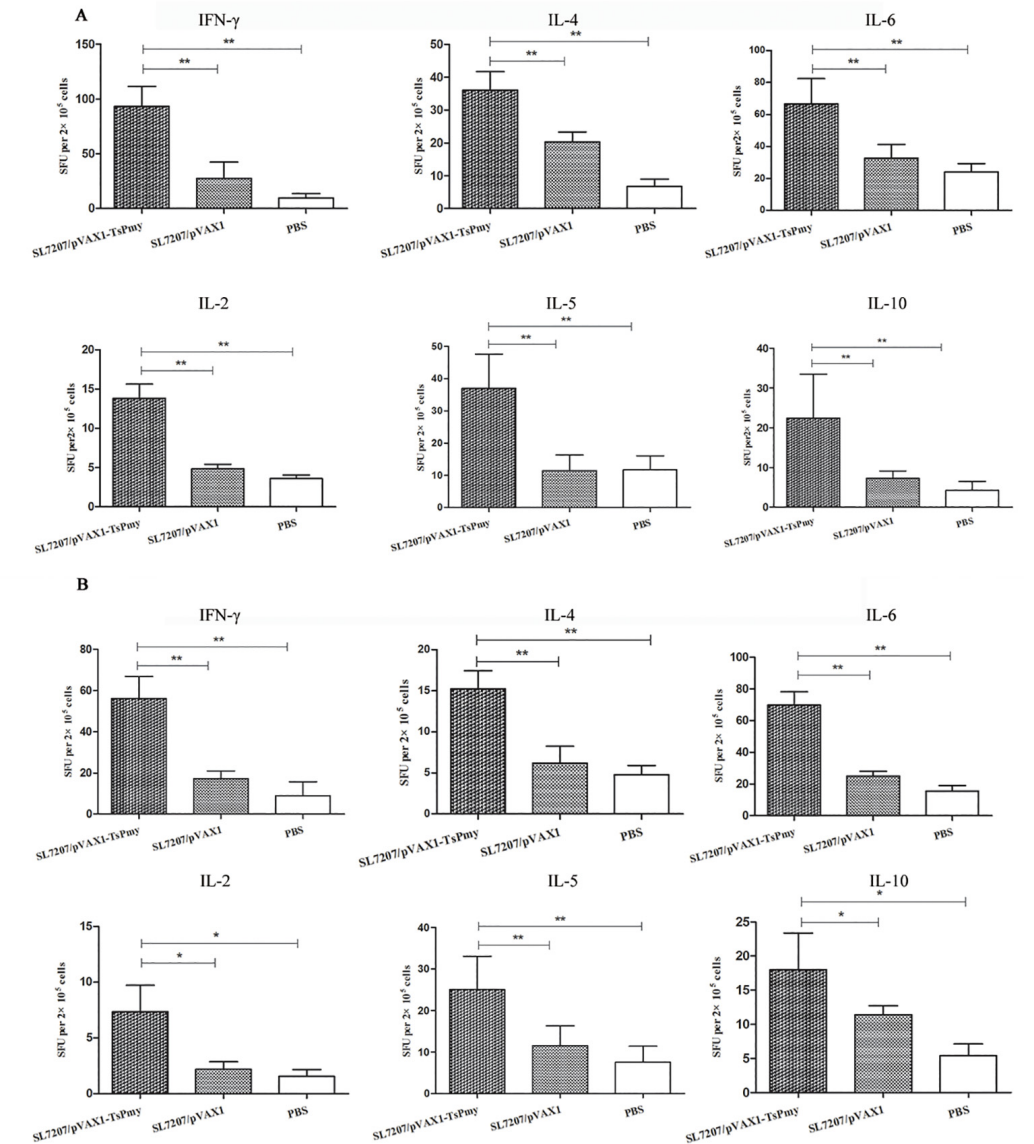
**Cytokine profile secreted by splenocytes (A) and MLN cells (B) upon r*Ts*Pmy stimulation.** Cytokine secretion was detected by ELISPOT assays after the cells were stimulated with r*Ts*Pmy for 48 h. The number of cytokine-producing cells is expressed as SFU/2×10^5^ cells per well after background subtraction. The results are the mean ± S.D. of cytokine levels (n = 5). Single asterisk (*) indicates significant differences (*p<*0.05) between two groups. Double asterisks (**) indicate significant differences (*p<*0.01) between two groups.

Compared with the SL7207/pVAX1 and PBS control groups, significantly increased levels of secretion of IFN-γ, IL-2, IL-4, IL-5, IL-6 and IL-10 were observed in both *Ts*Pmy-stimulated spleen (Fig 4A) and MLNs (Fig 4B) cells after vaccination, indicating that the Th1/Th2-mixed immune responses were significantly induced by the oral immunization of SL7207/pVAX1-*Ts*Pmy. Moreover, it also suggests that the immune response upon SL7207/pVAX1-*Ts*Pmy immunization occurred systemically (spleen) and locally in lymphocytes around the intestine (MLNs).

### Oral immunization with SL7207/pVAX1-*Ts*Pmy stimulates ASCs expressing CCR9 and CCR10 and IgA

**Fig 5:**
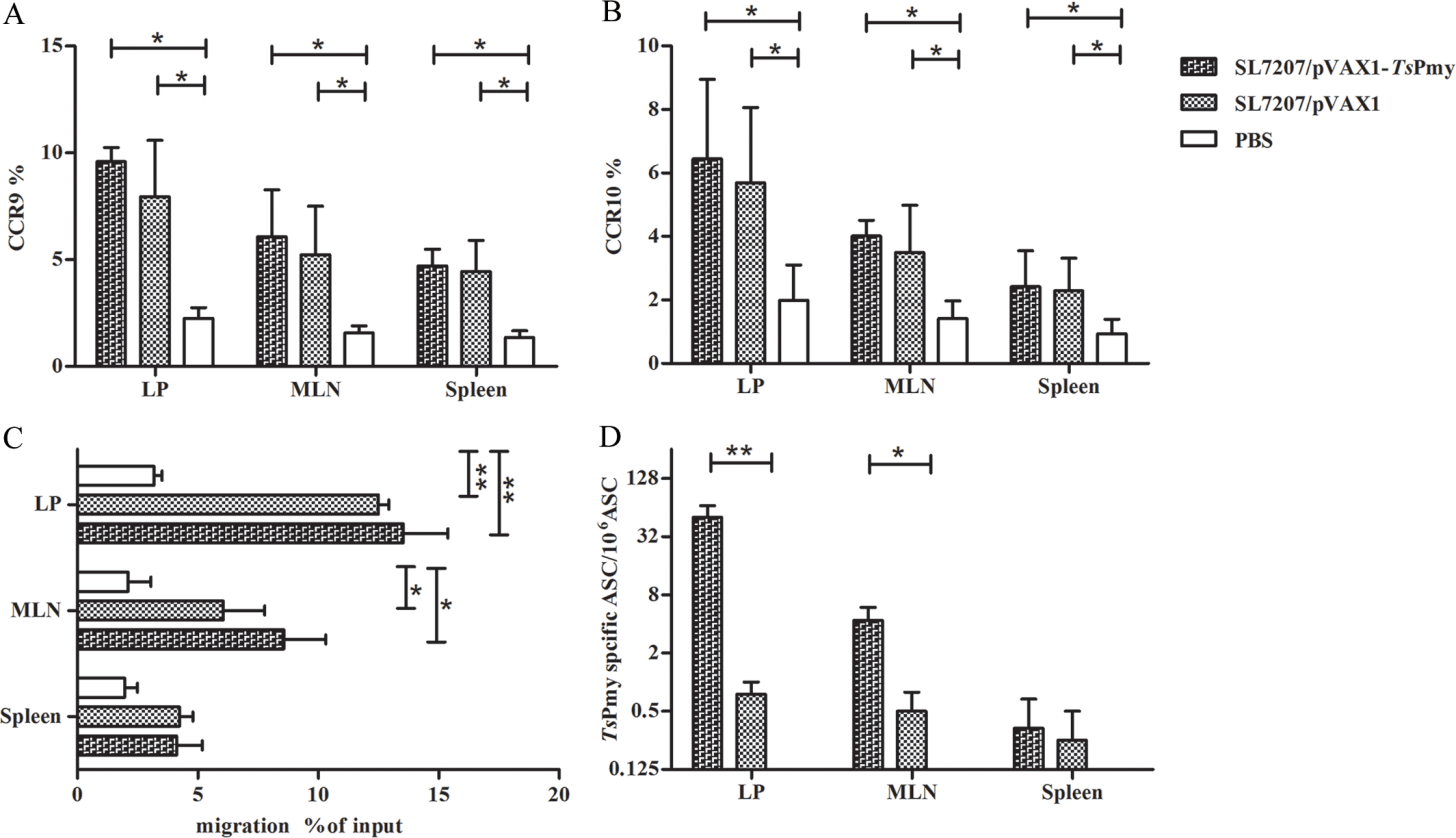
**Expression of CCR9 (A) and CCR10 (B) on ASCs isolated from the LP, MLNs and spleen of immunized mice were measured by flow cytometry.** The chemotaxis towards the chemokine CCL25 was measured for CCR9 expressed on ASCs isolated from LP, MLNs and spleen of mice using a Transwell incubator (C). The expression of anti-*Ts*Pmy IgA in migrated ASCs was measured with modified ELISPOT (D). The values shown for each group are the mean ± S.D. of antibody levels (n = 5). Single asterisk (*) indicates significant differences compared with its control mice (*p*<0.05). Double asterisks (**) indicates significant differences between two groups (*p*< 0.01).

To assess whether oral immunization of SL7207/pVAX1-*Ts*Pmy induces B lymphocytes into antibody secreting cells (ASCs), the total ASCs were isolated from the spleen, MLNs and intestinal *lamina propria* (LP) of mice immunized with SL7207/pVAX1-*Ts*Pmy three times. Both intestinal homing receptors CCR9 and CCR10 were highly expressed on ASCs from the spleen, MLNs and LP of mice immunized with both SL7207/pVAX1-*Ts*Pmy and SL7207/pVAX1 groups but not in the PBS group. However, the expression level of CCR9 and CCR10 was higher on LP than on MLNs and even less on spleen cells (Fig 5A and 5B). A similar phenomenon was also observed in the chemotaxis assay upon stimulation with CCL25, the chemokine ligand of CCR9. ASC cells isolated from the LP and MLNs of mice immunized with SL7207/pVAX1-*T*sPmy and SL7207/pVAX1 moved significantly more toward CCL25 than ASCs isolated from PBS control mice. The chemotaxis toward CCL25 was not significant in ASCs isolated from spleen immunized with SL7207/pVAX1-*Ts*Pmy and SL7207/pVAX1 compared to mice treated with PBS even though the levels of the former groups were higher than PBS (Fig 5C). Although we observed a higher expression of CCR9 and CCR10 on ASCs from mice immunized with a SL7207/pVAX1 empty control, similar to the level in mice immunized with SL7207/pVAX1-*Ts*Pmy, the antigen (*Ts*Pmy) specific IgA was only expressed in ASCs isolated from LP and MLNs of mice immunized with SL7207/pVAX1-*Ts*Pmy. No specific IgA was expressed in mice immunized with SL7207/pVAX1. The expression level of anti-*Ts*Pmy was higher in ASCs from LP than in MLNs without significant expression in the spleen (Fig 5D)

### Protective immunity against *T*. *spiralis* larval challenge

**Fig 6:**
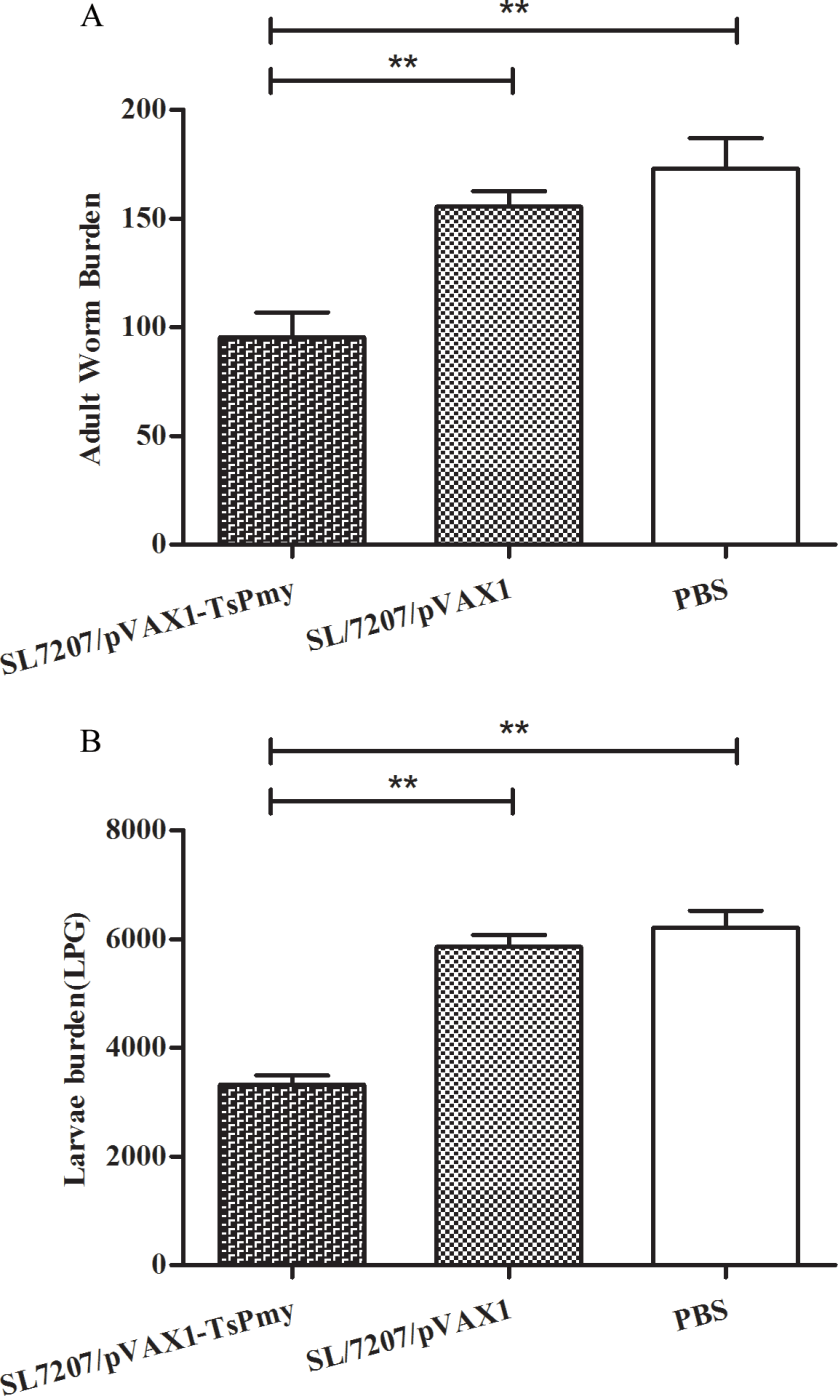
**The adult worm (A) and larvae per gram of muscle (LPG) burden (B) of vaccinated mice after challenge with 500 *T*.*spiralis* infective larvae.** The results are presented as the mean ± S.D. of 10 mice per group. Asterisks (**) indicate significant differences (*p<*0.01) between two groups.

The protective immunity was tested in immunized mice against *T*. *spiralis* larval challenge. The challenge results demonstrated that mice orally immunized with SL7207/pVAX1-*Ts*Pmy produced 44.8% reduction in muscle larvae burden (Fig 6A) and 46.6% reduction in adult worm burden (Fig 6B) after challenge with 500 *T*. *spiralis* infective larvae compared with the PBS control. The mice treated with SL7207/pVAX1 did not show any significant worm reduction compared to PBS control. The number of adult worms and ML collected from each group of mice was shown in S1 Table. This result demonstrated that oral immunization with *Ts*Pmy DNA vaccine delivered by attenuated *Salmonella* induced the partial protection against challenge infection with *T*. *spiralis* larvae.

## Discussion

Paramyosin is a thick myofibrillar protein found only in invertebrates [22]. *Ts*Pmy is the paramyosin expressed by *T*. *spiralis* that is not only a structural component of myofilament but also an immunomodulatory protein present on the surface of newborn larva and adult worm. *Ts*Pmy enables binding to C8/C9 [23, 24] and C1q [25] of the human complement, inhibiting classical complement activation and the formation of complement membrane attack complex (MAC), thereby protecting the parasite from attack by host activated complement. Immunization with r*Ts*Pmy protein[13, 23], immunogenic peptides [26, 27], or passive transfer of monoclonal antibody that specifically binds to the *Ts*Pmy C9 binding site[16] induced significant protection against *T*. *spiralis* larval challenge. Paramyosin in other helminths such as *Schistosoma mansoni*[28], *Brugia malayi*[29] and *Taenia solium*[30] also showed protective immunity against parasite infections in different animal models.

*Ts*Pmy is a large protein with 885 amino acids and a 102 kDa predicted molecular weight, which is difficult to express as a soluble recombinant protein [15]. The low expression yield and solubility hurdles its development as a recombinant protein vaccine for large scale production. DNA vaccine is considered an alternative, even optimal approach because of the simplicity of its manufacturing and distribution, biological stability and cost effectiveness [31]. DNA vaccines have been shown to induce protective immunity against abroad range of pathogens such as Dengue virus [32], intracellular protozoan *Leishmania major* [33] or helminth parasite *Schistosoma japonicum*[34]. DNA vaccine contains a eukaryotic expression vector that could improve protein folding, therefore enable surface-exposed epitopes to be correctly presented and enable post-translational modification [35]. Nevertheless, naked DNA vaccine has inefficient immunogenicity compared to protein vaccines, which need an appropriate delivery system to enhance the immune response. The attenuated *Salmonella* strain has been identified as a suitable vaccine vector that could deliver heterogeneous antigens to the gastrointestinal mucosa and other lymphoid tissues and enhance specific humoral, cell-mediated, and mucosal immune responses [36]. In our previous study, vaccination with *Ts*87 antigen delivered by attenuated *Salmonella* led to partial adult worm and muscle larva reduction following challenge with *T*. *spiralis* larvae [10]. In this study, we cloned *Ts*Pmy coding DNA into the eukaryotic expression vector pVAX1, and the recombinant pVAX1 containing *Ts*Pmy coding DNA was transformed into attenuated *S*. *typhimurium* SL7207 strain to form a bacteria-delivered *Ts*Pmy DNA vaccine. Mice orally vaccinated with attenuated *Salmonella-*delivered *Ts*Pmy DNA elicited significant protection against *T*. *spiralis* larval challenge in a murine model. The immunized mice showed a 44.8% reduction in adult worm burden in the intestines and a 46.6% reduction in larval burden in muscle tissues, which is the best protection we have ever obtained so far compared to vaccination with recombinant protein [10, 13] or peptide [26, 27] of *Ts*Pmy in our lab using the same mouse model (S2 Table).

Live attenuated *Salmonella* is an effective vector that can bring the target DNA to internal organs and lymph tissues efficiently. Only one week after oral inoculation of *Salmonella* carrying *Ts*Pmy DNA, *Ts*Pmy RNA transcription and protein expression were observed in the MLNs, liver and spleen. The strong deliverability and adjuvanticity of attenuated bacteria induced strong immune responses, including humoral and cellular immunity against targeting pathogen [37]. Indeed, mice orally vaccinated with attenuated *Salmonella*-delivered *Ts*Pmy DNA vaccine induced a high serum titer of anti-*Ts*Pmy IgG. A higher level of an IgG2a subclass than IgG1 detected in the sera of mice after the 2^nd^ immunization suggested that the vaccination induced a relatively mixed Th1/Th2 response, which was further confirmed by the cytokine profiles of splenocytes and MLNs that showed significant increases in both Th1 (IFN-γ, IL-2) and Th2 cytokines (IL-4, IL-5, IL-6 and IL-10) upon stimulation with r*Ts*Pmy. More importantly, live attenuated *Salmonella*-delivered *Ts*Pmy DNA vaccine was able to induce considerable secretion of antigen-specific sIgA in the intestinal mucosa, which plays a crucial role in the mucosal immunity against intestinal pathogens [38]. In addition to the strong Th1/Th2 mixed systemic immune responses, the sIgA-associated mucosa immunity induced by live bacteria-delivered DNA vaccine may contribute to the better protection against *T*. *spiralis* larval challenge in this study. Many lines of evidence support the notion that intestinal sIgA are secreted largely by plasma cells derived from B cells initially activated in gut-associated lymphoid tissue (GALT) after vaccination. After immunization, most naïve B cells migrate to the Peyer’s patches or MLNs and differentiate into plasma cells and return to the intestinal mucosa (*Lamina Propria*) to produce high-affinity sIgA [39, 40]. As we know, the epithelial cells of the small intestine express chemokine CCL25 and CCL28 that play important roles in mucosal immunity by recruiting IgA antibody-secreting cells (ASCs) that express their CCR9 and CCR10 receptors in the mucosal *lamina propria*[41]. In this study, we identified that the *Salmonella*-delivered *Ts*Pmy DNA vaccine immunized mice expressed CCR9 and CCR10, the receptor of chemokine CCL25 and CCL28, respectively, on the surface of ASCs isolated from the spleen, MLNs and LP. Indeed, the CCR9-expressing ASCs were able to migrate towards CCL25 by chemotaxis. Interestingly, more CCR9 and CCR10 expressing and antigen-specific IgA-expressing ASCs were observed in LP than in MLNs with the least in the spleen, indicating that more ASCs, especially *Ts*Pmy-specific IgA expressing ASCs, migrate towards intestinal lymphatic tissues being attracted by intestinal cells expressing CCT25/CCL28. Although the *Salmonella* bacteria themselves also stimulate the ASCs expressing CCR9 and CCR10 in the mice administered *Salmonella*/pVAX1 empty vector only reflected by the total IgA stimulation in this study (Fig 3A), only *Ts*Pmy-specific IgA secreted APCs existed in LP and MLNs.

In conclusion, our results demonstrated that oral immunization with attenuated *Salmonella*-delivered *Ts*Pmy DNA vaccine induced a mixed Th1/Th2 systemic immune response and a strong mucosal IgA response that protected mice from infection with *T*. *spiralis* with a 44.8% reduction in adult worm and a 46.6% reduction in muscle larva compared with the PBS control group. The expression of the homing receptors CCR9/CCR10 on antibody secreting B cells may be related to recruiting IgA-secreted B cells to local intestinal mucosa. Whether the *Salmonella*-delivered *Ts*Pmy DNA vaccine induced mucosal sIgA-mediated worm killing is under investigation. The attenuated *Salmonella*-delivered *Ts*Pmy DNA vaccine provides a feasible and promising approach for controlling trichinellosis in human and domestic animals.

## Supporting Information

S1 Table *T*. *spiralis* Adult worms and ML collected from each group of mice upon being challenged with 500ML each. (DOCX)

S2 Table The vaccine efficacy comparison among available *Ts*Pmy-based *T*. *spiralis* vaccines developed in our lab. (DOCX)
