# Supplementary material for: Oral Vaccination with Attenuated Salmonella typhimurium-Delivered TsPmy DNA Vaccine Elicits Protective Immunity against Trichinella spiralis in BALB/c Mice
Source: PLoS Negl Trop Dis. 2016 Sep 2;10(9):e0004952. doi: 10.1371/journal.pntd.0004952 (PMC5010209; doi:10.1371/journal.pntd.0004952)
Supplement: S1 Table — (DOCX) [file pntd.0004952.s001.docx]

S1 Table. *T. spiralis* Adult worms and ML collected from each group of mice upon being challenged with 500ML each.

| Group | Worm# | Mean±SD | Reduction% | ML#（LPG） | Mean±SD | Reduction% |
| --- | --- | --- | --- | --- | --- | --- |
| SL7207/pVAX1-*Ts*Pmy | 88,111,98, 90,112,77, 87,105,98, 88 | 95.4±11.4 | 44.8 %^**^ | 3307.5,2569.6,3213.7,2810.6,2955.0,3870.9,3947.8,2693.8,3852.7,3940.8 | 3316.2±550.4 | 46.6%^**^ |
| SL7207/pVAX1 | 160,152,157,168,157,157,146,145,150,162 | 155.4±7.2 | - | 6620.2,5071.9,5354.4,6110.4,5350.7,6745.3,5263.4,6115.4,5198.7,6772.3 | 5860.2±687.3 | - |
| PBS | 163,162,181,160,174,181,176,150,188,195 | 173.0±14.0 | - | 5221.5,5709.7,5219.7,5929.7,5668.3,7653.1, 6947.5,5117.7,7334.7,7328.7 | 6213.0±996.3 | - |

^**^*p*<0.01 compared to SL7207/pVAX1 or PBS controls.
