# Supplementary material for: Oral Vaccination with Attenuated Salmonella typhimurium-Delivered TsPmy DNA Vaccine Elicits Protective Immunity against Trichinella spiralis in BALB/c Mice
Source: PLoS Negl Trop Dis. 2016 Sep 2;10(9):e0004952. doi: 10.1371/journal.pntd.0004952 (PMC5010209; doi:10.1371/journal.pntd.0004952)
Supplement: S2 Table — (DOCX) [file pntd.0004952.s002.docx]

S2 Table. The vaccine efficacy comparison among available *Ts*Pmy-based *T. spiralis* vaccines developed in our lab.

| Vaccine name | Vaccine type | Adult worm reduction | ML reduction | Reference# |
| --- | --- | --- | --- | --- |
| *Ts*87DNA | DNA/*Salmonella* | 29.8% | 34.2% | 10 |
| *Ts*Pmy +ISA206 | Recombinant protein | - | 33.7% | 13 |
| *Ts*Pmy +ISA720 | Recombinant protein | - | 34.9% | 13 |
| *Ts*Pmy +Freund’s | Recombinant protein | - | 36.7% | 13 |
| *Ts*Pmy | Epitope (peptide) | - | 33.4% | 26 |
| *Ts*Pmy + *Ts*87 | Multi-epitopes (peptide) | - | 35.0% | 27 |
| *Ts*PmyDNA | DNA/*Salmonella* | 44.8% | 46.6% | Current study |
